# Live in vivo imaging of Egr-1 promoter activity during neonatal development, liver regeneration and wound healing

**DOI:** 10.1186/1471-213X-11-28

**Published:** 2011-05-20

**Authors:** Philipp Dussmann, Judith I Pagel, Sabina Vogel, Terese Magnusson, Rene Zimmermann, Ernst Wagner, Wolfgang Schaper, Manfred Ogris, Elisabeth Deindl

**Affiliations:** 1 Walter-Brendel-Centre of Experimental Medicine, Ludwig-Maximilians-University, Munich, Germany; 2 Max-Planck-Institute for Heart and Lung Research, Bad Nauheim, Germany; 3 Pharmaceutical Biotechnology, Center for System-based Drug Research, Department of Pharmacy, Ludwig-Maximilians-University, Munich, Germany; 4 Anaesthesiology, Intensive Care and Pain Therapy, University Clinic Frankfurt, Frankfurt, Germany

## Abstract

**Background:**

The zinc finger transcription factor Egr-1 (Early growth response 1) is central to several growth factors and represents an important activator of target genes not only involved in physiological processes like embryogenesis and neonatal development, but also in a variety of pathophysiological processes, for example atherosclerosis or cancer. Current options to investigate its transcription and activation *in vivo *are end-point measurements that do not provide insights into dynamic changes in the living organism.

**Results:**

We developed a transgenic mouse (Egr-1-luc) in which the luciferase reporter gene is under the control of the murine Egr-1 promoter providing a versatile tool to study the time course of Egr-1 activation *in vivo*. In neonatal mice, bioluminescence imaging revealed a high Egr-1 promoter activity reaching basal levels three weeks after birth with activity at snout, ears and paws. Using a model of partial hepatectomy we could show that Egr-1 promoter activity and Egr-1 mRNA levels were increased in the regenerating liver. In a model of wound healing, we demonstrated that Egr-1 promoter activity was upregulated at the site of injury.

**Conclusion:**

Taken together, we have developed a transgenic mouse model that allows real time *in vivo *imaging of the Egr-1 promoter activity. The ability to monitor and quantify Egr-1 activity in the living organism may facilitate a better understanding of Egr-1 function *in vivo*.

## Background

The transcription factor Egr-1 belongs to the Egr family (Egr-1 to -4) of zinc finger proteins [1,2]. The growth factor inducible gene was discovered after stimulation of neuronal cells with nerve growth factor (NGF) and therefore initially referred to as NGF inducible A (NGFI-A) [3]. Fibroblast growth factor 1 (FGF-1), platelet derived growth factor (PDGF), vascular endothelial growth factor (VEGF) and general serum proteins are also capable of activating Egr-1 (for a recent review see [4]). Egr-1 is an important activator of target genes such as angiopoetin 1 [5] or cell division cycle 20 gene (cdc20) [6], which in turn are key players in cell proliferation, migration and differentiation [5,6]. Furthermore, Egr-1 itself has been shown to promote haematopoietic cell differentiation towards the macrophage lineage [7,8]. Being in the crossfire of different growth signals makes Egr-1 an interesting candidate to be studied during embryogenesis and neonatal development [9]. In addition, Egr-1 has been associated with atherosclerosis [10], diabetes [11], wound healing [12] and tumor growth [13]. Although Egr-1 knockout mice are viable, liver regeneration after hepatectomy is decreased due to impaired progression of mitosis [6]. Hence, Egr-1 relates to various physiological and pathological processes. However, most of the gathered data on Egr-1 gene activation have been evaluated within *in vitro *studies and could not be confirmed when being re-evaluated in *in vivo *models [4]. For this reason, it is inevitable to study activation patterns in the living organism over time. In knockout mice, however, compensation of Egr-1 loss of function by other Egr family members cannot be excluded (Pagel et al, manuscript submitted). Since dynamic changes over time cannot be examined by end-point measurements, studying Egr-1 activity within the living organism could help in gaining new information on *in vivo *Egr-1 gene activation.

The firefly luciferase has been applied as a bioluminescent reporter in living mice using a photon imaging system for studying gene induction noninvasively [14]. We have established a transgenic mouse model using the murine Egr-1 promoter to control the expression of the luciferase reporter and utilized noninvasive bioluminescence imaging (BLI) to study the dynamics of Egr-1 gene activity in the same animal over time. This model was applied to analyze Egr-1 promoter driven luciferase expression during the development of neonatal mice between the ages of 7 to 21 days after birth, where we observed a continuous decrease in Egr-1 promoter activity over time within the examined areas (snout and paw). The activation pattern of Egr-1 during wound healing and tissue regeneration was followed in a model for wound healing of ear tissue and in liver regeneration after partial liver hepatectomy.

## Results & Discussion

**Figure 1:**
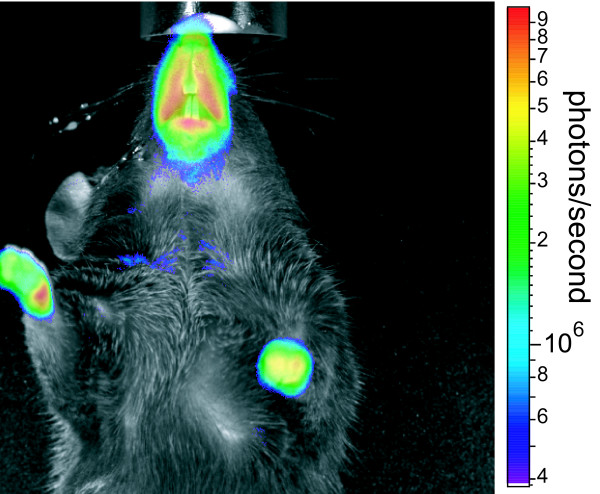
**Luciferase activity in adult Egr-1-luc mice**. Transgenic Egr-1-luc mice (4 month old, male or female) were anaesthetized with isofluorane in oxygen and received 6 mg luciferin in 100 μl PBS by i.p. injection. Ten minutes after injection BLI measurement was carried out (1 min signal collection, setting 'high resolution'). A representative animal is shown. The reflected light picture is overlaid by a a liacolor coded BLI image visualized in 'blend mode', which allows allocating the BLI signal to the respective areas shown in the underlying reflected light picture.

The Egr-1 promoter sequence was cloned into the plasmid pUHC13-2 replacing the CMV promoter in the CMV-Luc expression cassette. Transgenic mice were established by microinjecting the plasmid into male pronuclei of murine zygotes and transferred into pseudopregnant females (strain C57BL/6). For the Egr-1-luciferase (Egr-1-luc) construct seven founder animals were obtained. Breeding of founder animals with C57BL/6 wildtype mice led to an establishment of two lines (L1 and L2), which were further propagated. Egr-1-luc transgenic mice were viable and healthy and showed a normal life span, indicating no serious malformation due to the presence of the transgene. Breeding capabilities were also normal with a litter size of 5-8 pups. To show functional expression of luciferase, adult Egr-1-luc mice were injected with the luciferase substrate luciferin and the activity monitored in anaesthetized animals (Figure 1). In the living animal, highest signal intensities were observed in regions around the snout (especially lips), ears and paws, whereas in the fur covered regions the luciferase signal was not detectable, which could also be due to quenching effects. It can be postulated that these anatomical regions are still undergoing more developmental changes, i.e. cell proliferation than other areas of the body, such as the continued growth of the teeth. For example, Egr-1 has been identified to be involved in periodontal regeneration [15].

To analyze exemplarily whether primary cells from Egr-1-luc mice might also be suitable for *in vitro *investigations, we isolated vascular smooth muscle cells (SMC) from adult Egr-1-luc mice, cultured them and measured luciferase activity in cell lysates. On average, 4,000 RLU were measured per well (12-well plate, luc-negative cells give a background value of <300 RLU/well). This Egr-1 promoter activity in proliferating *in vitro *cultures of SMC is in line with Egr-1 activities described in the literature for SMC [16]. As the major aim of this work was to monitor Egr-1 activity *in vivo*, we did not further pursue *in vitro *cultures.

### *In vivo *monitoring of Egr-1 promoter activity during postnatal development and embryogenesis

**Figure 2:**
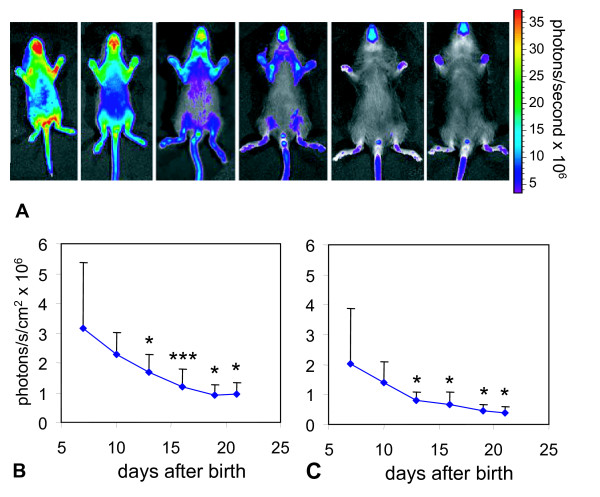
**Egr-1 promoter driven luciferase activity during postnatal development (day 7 - 21 after birth)**. Luciferase activity in Egr-1-luc mice at indicated age was measured by BLI after i.p. injection of luciferin. The luciferase signal was collected for 10 sec from the ventral side of the mice (n = 6).**A**: A reflected light picture of representative animals at indicated age is overlaid by a color coded BLI image. **B and C**: Luciferase activity was quantified within regions of interest (ROIs) placed at the paw (**B**) or snout area (**C**) and expressed as photons per second per cm^2 ^to correct for size differences in ROI size at different ages. A background ROI of similar size was subtracted. Median values of six animals + standard deviation are shown. * p < 0.05, *** p < 0.001; indicated day vs. day 7, Wilcoxon test

To monitor expression of the reporter during postnatal development, Egr-1-luc transgenic mice were imaged at day 7, 10, 13, 16, 19 and 21 after birth as described in "Methods". Day 7 was the earliest date, when intraperitoneal (i.p.) injections of the anesthetic and luciferase were tolerated. Animals from the same litter were measured for luciferase activity on indicated dates and were kept with the parents between the measurements to ensure feeding by lactating mother animals. For luciferase signal quantification we used defined regions of interest (ROI), which were placed over the snout and paw. Due to the lack of hair growth within those regions, which can otherwise reduce the luciferase signal and interfere with signal quantification, we did not expect a signal quenching. Other areas at the ventral side of the body were excluded from quantitative analysis, as due to the onset of hair growth, the BLI signal can be considerably quenched. The total number of photons collected per area was normalized to background levels by subtraction of the total counts per area measured in a similar sized ROI placed over a background area. As shown in Figure 2A, at day 7 after birth mice showed strong luciferase activity throughout the entire ventral side of the body. A clear reduction of luciferase activity within the areas of paws, snout, ears and tail was observed during their development throughout the following 2 weeks. All other areas, where the onset of fur growth leads to signal quenching, were excluded from further analysis. When quantifying the luciferase signal in ROIs at paw (Figure 2B) and snout (Figure 2C), luciferase activity was found to be reduced over time reaching 30% or 40%, respectively, at day 21 compared to the value at day 7. To investigate, whether there is remaining Egr-1-luc activity in fur-covered regions of adult mice, an adult mouse was sacrificed ten minutes after luciferase injection, the body cavity opened and the skin partially removed from the ventral area. Immediately thereafter BLI analysis was started (additional file 1). Besides Egr-1-luc activity at the snout area (as already shown in Figures 1 and 2A), there was remaining activity within the exposed skin, but not in other organs like liver or in muscle tissue. Hence, for multiple BLI measurements over time, rather superficial areas, like skin can be analyzed in living animals, whereas low luciferase signals within internal organs might be outshined by the skin activity of Egr-1 driven luciferase.

**Figure 3:**
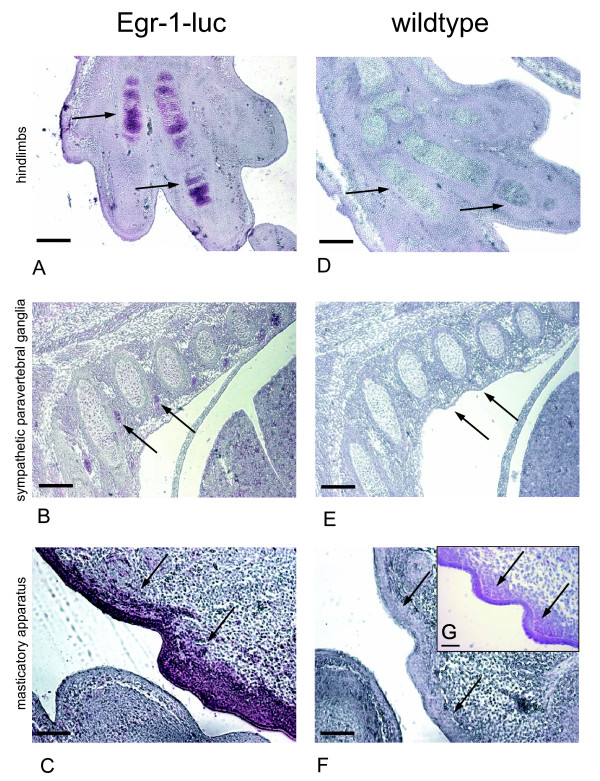
**Immunohistochemical analyses of luciferase and Egr-1 expression during embryonic development**. Egr-1-luc (**A**, **B**, **C **and **G**) and wildtype (**D**, **E **and **F**) C57BL/6 embryos on day E14 of development were stained for luciferase protein (**A-F**) or Egr-1 protein (**G**). In bone primordia of hindlimbs (**A**), sympathetic paravertebral ganglia (**B**) and masticatory apparatus (**C**) luciferase positive areas (arrows) are stained in lilac in transgenic embryos, in wildtype embryos no luciferase signal was detected (**D-F**). When staining for Egr-1 protein, a similar pattern of protein expression was found - exemplarily shown for the masticatory apparatus (**G**) - as for luciferase (**C**); scale bar: 50 μm

We also evaluated Egr-1 promoter activity in transgenic and nontransgenic embryos on day E14 of development (Theiler Stage 22) by immunohistochemical analysis of luciferase and Egr-1 (Figure 3). In accordance with the BLI data, bone primordia of hind limbs stained positive for luciferase (Figure 3A). Various neuronal structures demonstrated luciferase staining, among them the sympathetic paravertebral ganglia (Figure 3B). Furthermore, an intense immunoreactivity was detected at the masticatory apparatus, especially at the area of the palatal shell (Figure 3C); snout and whisker follicles showed only weak staining (data not shown). Wildtype (non-transgenic) animals did not display any positive staining (Figure 3D-F). When staining sections with Egr-1 antibody, a pattern corresponding to the previously described endogenous Egr-1 expression [9], was observed. The strong luciferase positive staining at the palatal shell of the masticatory apparatus, which has not been described before, could also be well correlated to Egr-1 expression (Figure 3G). The pattern of Egr-1 promoter activity (developing limbs, central nervous system, mandibles) is at least in part similar to the pattern of ERK signaling during embryogenesis, where major sites of ERK activity were observed, besides others, in limb buds, forebrain and the frontonasal process [17], which points at the interconnection between ERK and Egr-1 signaling. Our data clearly demonstrate that Egr-1 is highly upregulated throughout the body during neonatal development, where we observed maximal activity at day seven after birth, the earliest time point measured (Figure 2). Similar observations were made with transgenic mice expressing vascular endothelial growth factor promoter driven luciferase in neonatal mice [18], and maximal activity was observed one week after birth followed by a continuous decline in activity. When stimulating endothelial cells *in vitro *with VEGF, TNFα or thrombin, activation of Egr-1 was observed, although the induction of Egr-3 was more profound [19]. Hence, we postulate that there appears to be a spatial and temporal correlation between VEGF and Egr-1 activity during neonatal development. VEGFR-2 promoter controlled luciferase expression was analyzed in a similar way in neonatal mice [20]: at the earliest time point measured (2 weeks after birth) highest luciferase activity was found throughout the entire body, whereas in 6 week old mice the signal was about 100-fold reduced and remained constant for up to 15 weeks after birth. In our study, Egr-1 promoter controlled luciferase activity reached baseline levels already three weeks after birth indicating a faster decrease compared to VEGFR-2. *In vitro *studies showed the interconnection of VEGF and Egr-1, as in endothelial cells VEGF stimulation led to initially high Egr-1 levels [21]. Besides VEGF, other growth factors upregulated in neonatal organisms, such as FGF-1 and -2 can activate Egr-1 [22], whereas the decrease in Egr-1 activity can be explained by the negative feedback loop of Egr-1, which can bind to its own promoter [21] leading to a 'fine tuning' of its activity. Apparently Egr-1 is involved in the cell proliferation process during postnatal growth. The activity of Egr-1 is on a high level almost all over the entire ventral anatomy of the neonatal mouse and decreases until reaching a baseline activity when mice are fully grown. During this stage, the only significant Egr-1 activity was observed at the paws, snout, ears, and tail, which is in line with observations made while measuring VEGFR-2 promoter driven luciferase [20]. It still has to be determined, which specific processes are taking place on these sites of high Egr-1 promoter activity relative to the rest of the body.

### Egr-1 activation in regenerating liver

**Figure 4:**
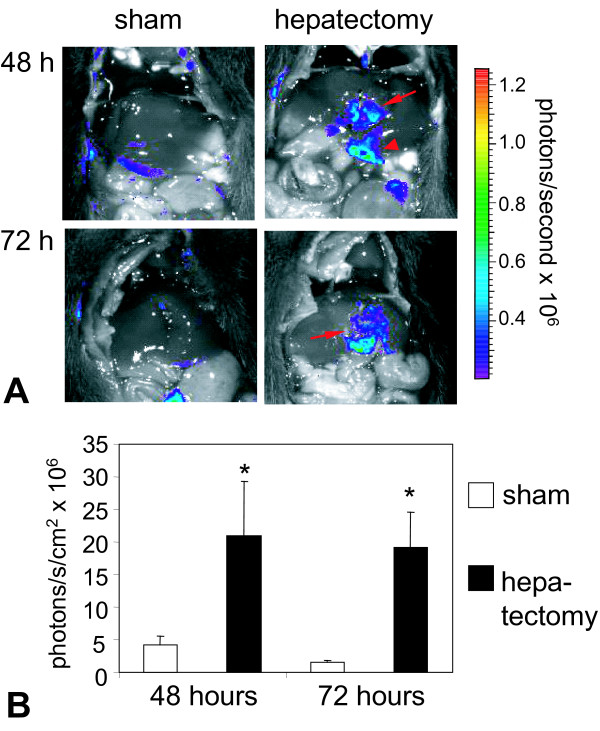
**Egr-1 promoter driven luciferase activity after partial liver hepatectomy**. **A**: BLI of sham operated (left) or hepatectomized (right) Egr-1-luc mice at 48 h (top row) and 72 h (bottom row) after surgery. Representative animals from n = 3-6 are shown. Red arrows denote the site of initial surgery, arrowhead points at the edge of a liver lobe showing luciferase activity. **B**: Quantitative luciferase signals from ROIs placed over the liver area of sham operated or hepatectomized mice 48 h or 72 h after surgery. A representative background ROI of comparable size was subtracted to account for background activity. n = 3-6; mean values of six animals + standard deviation are shown. *p < 0.05 sham vs. hepatectomy (U-test, Mann-Whitney).

**Figure 5:**
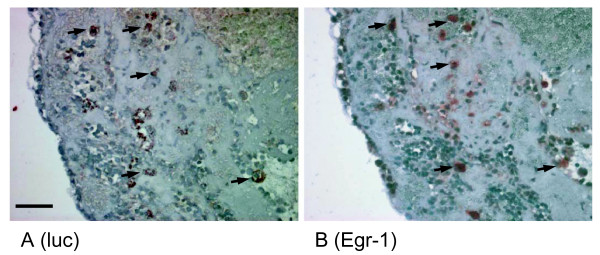
**Immunohistochemical analyses of Egr-1 driven luciferase expression in regenerating liver**. Egr-1-luc mice 4-8 weeks of age were subjected to one third liver hepatectomy as described in "Methods". Forty-eight hours after surgery mice were sacrificed, liver tissue fixed in PFA and stained for luciferase (luc). Tissue next to the site of surgery (rim upper left corner in both images) is shown and cells staining positive for luciferase (**A**) as well as Egr-1 (**B**) appear as clusters with brown-reddish staining (arrows; scale bar: 50 μm)

Liver hepatectomy in rodents leads to induction of cell division in the majority of hepatocytes 1-2 days after surgery [23,24]. Here we used *in vivo *BLI to monitor the activity of the Egr-1 promoter in the transgenic Egr-1-luc mice 48 h (n = 6 and n = 3 control) and 72 h (n = 4 and n = 3 control) following 1/3 hepatectomy. For quantification, ROIs were placed over the areas of regenerating liver tissue close to the primary excision site. The highest Egr-1 activity was observed at regions directly adjacent to the original sectioning wound with some elevated activity at the edges of the liver lobes, as shown in Figure 4A. When quantifying the BLI signal within the area next to the excision site, an up to 12-fold signal increase was observed compared to sham operated animals, both at 48 h and 72 h after surgery (Figure 4B). After hepatectomy, clusters of cells stained positive for luciferase (Figure 5A) and for Egr-1 (Figure 5B) in a similar pattern in sections of liver tissue at the site of surgery.

**Figure 6:**
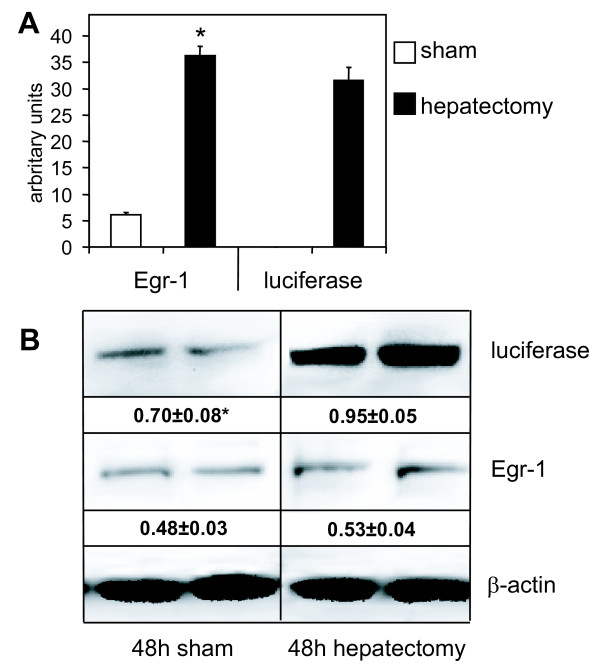
**mRNA and protein levels of Egr-1 and luciferase after partial hepatectomy**. Egr-1-luc mice were subjected to one third hepatectomy or sham operation, sacrificed 12 h (**A**) or 48 h (**B**) after surgery and liver tissue subjected to mRNA analyses by qRT-PCR (**A**) or Western blot analyzing protein levels of Egr-1 and luciferase (**B**). **A**: mRNA levels of Egr-1 and luciferase, respectively (average relative mRNA levels relative to 18S rRNA levels); mRNA levels of luciferase in sham operated animals were below the detection limit. n = 4, mean values + standard deviation are shown; *p < 0.05 sham vs. hepatectomy (U-test, Mann-Whitney).** B**: Representative Western blots showing the protein levels of Egr-1, luciferase and β-actin for sham operated (left panel) or hepatectomized animals (right panel), respectively; data from two representative animals per treatment are shown. Numbers indicate relative luciferase and Egr-1 expression calculated from optical densities (OD) of luciferase, Egr-1 and ß-actin protein bands. n = 4, mean values + standard deviation are shown; *p < 0.05 sham vs. hepatectomy (U-test, Mann-Whitney).

To obtain a quantitative view on Egr-1 levels during liver regeneration, animals were sacrificed 12 h or two days after surgery and liver tissue homogenized for mRNA and protein quantification of Egr-1 and luciferase, respectively (Figure 6). Twelve hours after surgery, mRNA levels of Egr-1 were six times higher compared to the sham operated group (Figure 6A). For luciferase, the mRNA level was not detectable in the sham operated group, whereas in hepatectomized mice, a strong signal was found. Analyzing protein levels two days after surgery, an increased signal was found for both Egr-1 and luciferase (Figure 6B). Compared to Egr-1 this increase was more pronounced for luciferase, which can be explained by the fact that luciferase protein has a considerably longer intracellular half live (3 hours, [25]) than Egr-1 protein, which is more rapidly degraded (half live <2 h, [26]). The involvement of Egr-1 in liver regeneration has been first described by Mueller and colleagues [27], and Egr-1^-/- ^mice showed significantly delayed liver regeneration after hepatectomy [6]. The importance of Egr-1 expression in liver injury has also been described in ethanol induced fatty liver, where Egr-1 promoted TNFα expression [28]. In our liver regeneration experiments, we have demonstrated that luciferin expression was induced at the early stages of liver regeneration. Already 12 h after surgery, mRNA levels of luciferase and Egr-1 were strongly elevated (Figure 6), which is in line with results obtained in rats [27]. Interestingly, luciferase activity and protein levels were elevated both at 48 h and 72 h after surgery (Figures 4 and 6). During the regeneration of liver tissue, quiescent liver cells re-enter the cell cycle and divide until the original liver mass is restored [23]. Besides induction of proliferation, partial hepatectomy can lead to local hypoxia and upregulation of hypoxia inducible factor 1α (HIF-1α) [29]: peak levels of HIF-1α were observed at 24 h after surgery, whereas peak levels of VEGF appeared at later time points. Hence, the induction of Egr-1 after partial hepatectomy can be at least in part due to hypoxic conditions, an effect already described for macrophages *in vitro*, where hypoxia induced Egr-1 expression occurred [30]. Even though the major Egr-1 promoter activity was observed in the area of the initial surgery wound, which is mainly due to the wound healing process, some elevated activity was seen at the edges of the lobes suggesting the onset tissue regeneration by the means of cell division and proliferation. These results support the reported findings on impaired mitosis in Egr-1 deficient mice [6] and evidence that Egr-1 is not only induced within the healing process, but also during other processes where cell division and proliferation is involved.

### Egr-1 activation in wound healing

**Figure 7:**
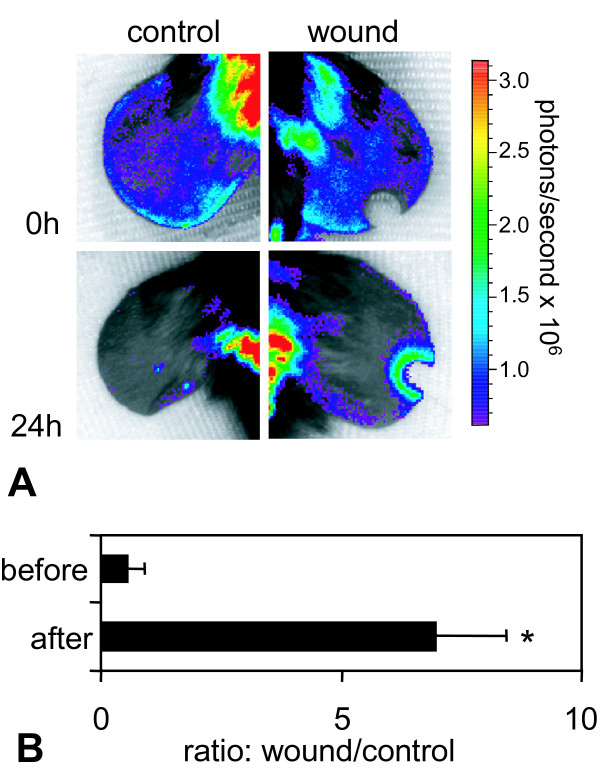
***In vivo *bioluminescence imaging of the ear wound**. In Egr-1-luc mice between the ages of 4-8 weeks an ear wound was inflicted in one ear. Twenty-four hours after infliction animals were subjected to BLI. Luciferase signal was collected for 2 min from the wounded or the control ear, respectively. **A**: Color coded BLI image overlaid onto a reflected light image from the control ear (left) or wounded ear (right) immediately (top row) or 24 h (bottom row) after wound infliction. **B**: Quantitative luciferase signals from ROIs placed over the wound site or a similar sized ROI at the control ear. n = 3, mean values + standard deviation are shown; *p < 0.05 control vs. wound (U-test, Mann-Whitney).

Using *in vivo *BLI, we monitored the activity of the Egr-1 promoter in Egr-1-luc mice immediately after the infliction of a punch wound on the right ear. For quantification we used defined ROIs (please note: color scale in Figure 7 is on an approx. 10-fold lower level than in Figure 2A). The Egr-1 activity showed a major increase in the immediate vicinity surrounding the wound, while the more distant areas did not show any difference compared to the control ear (Figure 7A). Placing a ROI over the area surrounding the wound, a >12-fold increased BLI signal was found 24 h after infliction of the wound when compared to a similar sized ROI on the adjacent control ear (Figure 7B). At the early stages of wound healing, an inflammatory response followed by re-epithelialization of the wound area and establishment of granulation tissue with accompanying neovascularization occurs [31]. Due to the interaction of Egr-1 and inflammatory cytokines, like TNFα [28,32] and others, Egr-1 is involved in the wound healing process. In Egr-1 null mice, wound healing and the influx of inflammatory cells was shown to be significantly reduced, whereas Egr-1 over expression led to exuberant tissue repair and enhanced collagen production [12]. Our data of the ear wound experiment suggest that Egr-1 plays a substantial role in the wound healing process, as its activity increased around the immediate wound area.

## Conclusion

In summary, the present study followed the Egr-1 activation pattern over time in the transgenic Egr-1-luc animal model and showed the spatial expression patterns and their time dependent changes *in vivo*. This transgenic mouse provides a convenient model for studying Egr-1 expression during neonatal development and wound healing at areas were the fur of mice with C57Bl/6 background does not interfere with BLI imaging. Monitoring Egr-1 activity within internal organs, such as in the liver regeneration model presented, was only possible by endpoint measurements with animals having an opened body cavity. To further improve its usability for BLI, cross-breeding into hairless mice will improve its sensitivity. Moreover, it will then offer a useful tool for monitoring effects of pharmaceutical drugs over time *in vivo*.

## Methods

### Transgenic mice (Egr-1-luc)

#### Cloning strategy

The vector containing the murine Egr-1 promoter was a generous gift from Martin Braddock (Glaxo Wellcome, United Kingdom). From this vector, the Egr-1 promoter [33] compassing the sequence from -930 to +237 base pairs relative to the Egr-1 promoter transcriptional start site [34,35] was isolated by SalI restriction. 5'ends were filled-in with DNA polymerase I (Klenow enzyme) and cloned into the pUHC13-2 vector by blunt end ligation thereby replacing the CMV promoter. The pUHC13-2 vector, which was a generous gift from H. Bujard (ZMBH, Germany), is a derivate of pUHD10-1 [36] and was originally developed by U. Baron in the laboratory of H. Bujard. In short, the reporter plasmid pUHC13-2 containing the promoter-enhancer sequence of the CMV promoter followed by a polylinker and the luciferase gene of *Photinus pyralis *(firefly) fused to the SV40 small-t intron and poly(A) signal was digested with HindIII and XhoI to excise the CMV promoter. The 5'ends were filled in with Klenow enzyme and ends were dephosphorylated with alkaline phosphatase. After cloning the Egr-1 promoter into pUHC13-2 vector, the Egr-1 promoter - luciferase reporter gene - SV40 small-t intron fragment was isolated by AseI and PvuI digestion. Finally the transgene was purified using a QIAquick Gel Extraction Kit (Qiagen). All constructs obtained were reviewed and verified by sequencing.

#### Establishing Egr-1-luc transgenic mouse lines

Egr-1-luc transgenic mice were established by micro-injecting 2 pl of the transgene (5 ng/μl) into male pronuclei (identified by size) of murine zygotes and transferred into pseudopregnant females (strain C57BL/6). The presence of the transgene was confirmed by means of PCR using a specific primer combination spanning the region between the reporter gene luciferase and the SV40 small-t intron (forward primer: 5'- GAG ATC GTG GAT TAC GTC GC - 3'; reverse primer: 5'- TGC TCC CAT TCA TCA GTT CC -3').

### In vivo imaging of luciferase activity

Animals were housed in individually vented cages with a 12 h day/night cycle and chow and water provided *ad libitum*. All animal procedures were approved and controlled by the local ethics committee and carried out according to the guidelines with the German law for protection of animal life.

*In vivo *imaging was performed using the IVIS Lumina Imaging System (Caliper Life Sciences GmbH) as recently described [37]. For the developmental studies, Egr-1-luc transgenic mice were anesthetized by i.p. injection of xylazin/ketamin (0.375 ml/0.635 ml in PBS, respectively); for liver regeneration and wound healing studies animals were anaesthetized with 2.5% isofluorane in oxygen. Ten minutes after i.p. injection of 300 mg/kg luciferin (Promega, Hilden, Germany) the bioluminescence signal was collected for one to three minutes. Reflected light pictures were taken during illumination with four white LED. Image acquisition and processing was carried out using Living Image 2.60.1 - IGOR Pro 4.09 Software.

### Immunohistochemistry

For immunhistochemical detection of luciferase and Egr-1 in liver, the tissue was fixed in 4% paraformaldehyde (PFA) over night at 4°C and subsequently embedded in paraffin. Embryos (n = 6, littermates) were collected at day 14 of development for detection of luciferase and Egr-1, fixed in 4% PFA for three days and placed in a solution of Na_4_EDTA (ethylenediaminetetraacetic acid tetrasodium salt), 200 g/L, pH 7.1 (adjusted using 20% w/v citric acid) for decalcification before being embedded. Four μm sections were mounted on Super Frost^® ^Plus slides (Thermo Scientific Gerhard Menzel). Antigen retrieval for luciferase staining was achieved with Pronase E (Sigma-Aldrich) diluted in 0.5 M Tris buffer (0.1% w/v) for 20 min at room temperature; Egr-1 antigen retrieval was performed in a steamer with sodium citrate buffer (10 mM sodium citrate, pH 6.0) for 20 min. Endogenous peroxidase activity was quenched by treatment with 1% H_2_O_2 _for 30 min. Slides were incubated over night at 4°C with an anti-luciferase goat polyclonal horseradish peroxidase (HRP) conjugated antibody (Abcam, 1:50 in Tris-buffered saline (TBS)/0.3% BSA (TBS-B)) and an Egr-1 rabbit monoclonal antibody (clone: 15F7, # 4153, Cell Signaling, dilution 1:50 in TBS-B), respectively. For Egr-1 staining, a biotinylated secondary anti-rabbit antibody was prepared using a rabbit ABC kit (VECTASTAIN^® ^Elite ABC system, Vector). Immunoreactivity was visualized with the chromogen 3-amino-9-ethyl-carbazole (AEC) (AEC single solution, Invitrogen) for 20 min.

### One-third liver hepatectomy

Hepatectomy was carried out based on a currently published protocol [24] with slight modifications. In brief, mice were anaesthetized with isoflurane and 50 μl carprofen given i.p. for pain reduction. All surgical steps were carried out as described [24], except that only the median lobe was resected leaving a small ischemic stump behind. After surgery, mice received daily 50 μl carprofen i.p.. In sham operated animals, only the midline incision was performed and sutured. At indicated time points after surgery, animals were anaesthetized, the liver was exposed after luciferin injection and images of the ventral view of the fully exposed liver were collected.

### Ear wound healing model

A punch wound of approximately 1.5 mm in diameter was inflicted with an ear notcher on one ear of Egr-1-luc mice according to the standard procedure of animal labeling. At indicated time points after the wound setting, BLI from the ear region was carried out as described above, only that the ear was immobilized with adhesive tape during imaging. As a control, the untreated ear was measured.

### Cell culture

Primary cultures of murine aortic SMC were established as previously described [38] and cultured on gelatin-coated plates with standard HAM's F12/Waymouth 1:1 medium (Biochrom), 10% FCS (fetal calf serum) and antibiotics (100 U/ml penicillin, 100 μg/ml streptomycin, 0.25 μg/ml amphotericin B). For stimulation, cells between passages three and four were serum starved for two days. Luciferase quantification was performed in cell lysates as described [39]. In brief, an aliquot of cell lysate was quantified in a tube luminometer after injection of substrate solution for 10 sec, background values from wildtype cells were deducted from the measurement; two nanogram recombinant luciferase (Promega, Mannheim, Germany) correspond to 10^7 ^relative light units (RLU).

### RNA isolation and quantitative Real Time PCR (qRT-PCR)

Total RNA was isolated according to the procedure of Chromzynski and Sacchi [40] from frozen liver samples isolated 12 h after hepatectomy or sham operation (n = 4). One microgram of DNase treated total RNA was reverse transcribed using random nonamers (Roche) and a 1st Strand cDNA Synthesis Kit for RT-PCR (Roche). qRT-PCR was performed with a Light Cycler 1.5 (Roche) in a reaction volume of 10 μl using a Light Cycler^® ^FastStart DNA Master^Plus ^SYBR Green I Kit (Roche) and 50 pmol of each primer (Egr-1, forward: 5'- CGA ACA ACC CTA TGA GCA CCT G - 3'; reverse: 5'- CAG AGG AAG ACG ATG AAG CAG C - 3'; luciferase, forward: 5'- CAG ATG CAC ATA TCG AGG TG - 3'; reverse: 5'- CAT ACT GTT GAG CAA TTC ACG - 3'; 18S rRNA, forward: 5'- GGA CAG GAT TGA CAG ATT GAT AG - 3'; reverse: 5'- CTC GTT CGT TAT CGG AAT TAA C - 3'). Three independent qRT-PCR reactions were performed on each template. An initial denaturation step at 95°C for 10 min was followed by 40 cycles of denaturation (95°C, 10 sec), annealing (64°C for Egr-1; 58°C for luciferase, 64°C for 18S rRNA, 5 sec), and extension (72°C, 15 sec). Melt curve analyses were performed to control specific amplification. Results were normalized to the expression levels of the 18S rRNA.

### Western blot

Protein extracts of liver tissue samples were isolated 48 h after hepatectomy as described [41]. Equal amounts of protein were separated on a 4-20% Tris-glycine gel (Serva) and immunoreactive bands were visualized using Super-Signal-Femto-West (Pierce) with a HRP conjugated rabbit polyclonal antibody against firefly luciferase (1:1000, Santa Cruz Biotechnology), a rabbit monoclonal antibody against Egr-1 (1:500, Cell Signaling) or β-actin (1:2000, Sigma), respectively. Luminescence was evaluated using Hamamatsu 1394 ORCA-ERA camera, AequoriaMDSTM Macroscopic Imaging System and Wasabi software (Hamamatsu Photonics, Herrsching, Germany). Protein bands were quantified by densitometry, and results expressed as Luc/ß-actin and Egr-1/ß-actin ratio, respectively. For negative control, the first antibody was omitted. Blots were repeated at least twice.

### Statistics

Statistical analyses were performed using WinStat. p-values <0.05 were regarded as statistical significant and calculated using either the non-parametric U-test (according to Mann-Whitney) or the Wilcoxon test.

## Abbreviations

Egr-1: Early growth response 1; Luc: luciferase; CMV: cytomegalovirus; SMC: vascular smooth muscle cells; RLU: relative light units; i.p.: intraperitoneal; ROI: region of interest; BLI: bioluminescence imaging

## Authors' contributions

PD carried out all bioluminescence experiments and surgical procedures and drafted the manuscript; JIP carried out the histological studies, qRT-PCR analyses, Western blot analyses and was involved in writing the manuscript; SV generated the transgenic mice; TM carried out the *in vitro *studies with Egr-1-luc expressing smooth muscle cells; RZ was involved in Egr-1-luc strain development; WS and EW were involved in discussions; MO and ED designed the study, coordinated the project, drafted and finalized the manuscript. All authors read and approved the final manuscript.

## Supplementary Material

Additional File 1 **BLI of adult Egr-1-luc mice with opened body cavity**. Transgenic Egr-1-luc mice (one month old) received 6 mg luciferin in 100 μl PBS by intraperitoneal injection. Ten minutes thereafter the animal was killed by cervical dislocation, the body cavity opened immediately, skin from the ventral side partially removed and BLI measurement was carried out (10 min signal collection, setting 'high resolution'). A representative animal is shown with similar amplification setting as in Figure 2A. Click here for file
